# Potential Utility of ^123^I-MIBG Scintigraphy as a Predictor of Falls in Parkinson's Disease

**DOI:** 10.3389/fneur.2019.00376

**Published:** 2019-04-12

**Authors:** Nagahisa Murakami, Wataru Sako, Shotaro Haji, Takahiro Furukawa, Yoichi Otomi, Hideki Otsuka, Yuishin Izumi, Masafumi Harada, Ryuji Kaji

**Affiliations:** ^1^Department of Clinical Neuroscience, Institute of Biomedical Sciences, Tokushima University Graduate School, Tokushima, Japan; ^2^Department of Radiology, Institute of Biomedical Sciences, Tokushima University Graduate School, Tokushima, Japan; ^3^Department of Medical Imaging/Nuclear Medicine, Institute of Biomedical Sciences, Tokushima University Graduate School, Tokushima, Japan

**Keywords:** Parkinson's disease, falls, MIBG, prognosis, motor symptom

## Abstract

**Background:** Falls are associated with poor prognosis in patients with Parkinson's disease (PD). Although several factors related to falls were reported in patients with PD, objective predictors of falls are not identified. We aimed to determine whether ^123^I-meta-iodobenzylguanidine (MIBG) cardiac scintigraphy could be a useful biomarker to predict falls.

**Methods:** Forty-five patients with PD were enrolled in this study. These subjects were followed up more than 5 years after MIBG scintigraphy and were divided into two groups: one with decreased uptake of MIBG and the other without decreased uptake of MIBG. The cut-off value for the delayed heart-to-mediastinum ratio was 1.8. Kaplan-Meier analysis and a log-rank test were performed to test the predictive power of MIBG cardiac scintigraphy for falls. Univariate analysis was selected because we did not have appropriate data for adjustment, such as motor and cognitive assessment.

**Results:** The group with decreased uptake of MIBG had a significantly higher incidence of falls than that without decreased uptake of MIBG (*P* = 0.022, log-rank test).

**Conclusions:** Although the limitations of this study were lack of several key factors including motor and cognitive assessment, MIBG cardiac scintigraphy may be used to predict falls in patients with PD.

## Introduction

Falls predict an unfavorable prognosis due to poor motor function in patients with Parkinson's disease (PD), which are attributed to considerable factors including motor and non-motor impairments (1). Although it is not easy to overcome falls, several interventions have been developed. These include administration of acetylcholinesterase inhibitors (2) or the prodrug of epinephrine (3), and Tai chi (4) or other types of exercise (5). Early identification of patients at high risk for falls is essential to ensure that the above interventions are undertaken before the patient is bed-ridden due to fall-related injury. There is thus a need for the identification of risk factors that allow prediction of falls.

In terms of a risk factor for a poor prognosis, ^123^I-meta-iodobenzylguanidine (MIBG) cardiac scintigraphy has been reported to be a predictor of dementia in PD (6). Patients with PD tend to have reduced uptake of MIBG, although this measure varies widely (7). We hypothesized that the wide-range of MIBG uptake might help define subgroups of patients with PD with difference in prognosis. To test our hypothesis, we investigated the relationship between MIBG uptake and falls using data from 45 patients with PD at the Tokushima University Hospital.

## Materials and Methods

### Participants

A total of 438 patients with PD were identified from the medical records of inpatients and outpatients at the Tokushima University Hospital between April 1st, 2007 and September 30th, 2017. Ultimately, 45 patients who were diagnosed with clinically established or probable PD according to the internationally established PD criteria (8), and were followed up for more than 5 years after MIBG evaluation were included in this study. These patients were divided into two groups: one with decreased uptake of MIBG [delayed heart-to-mediastinum ratio [H/M ratio] < 1.8], and the other without decreased uptake of MIBG (delayed H/M ratio ≥ 1.8). We considered the following variables: sex, age at onset, age at MIBG evaluation, disease duration, Hoehn-Yahr stage, daily levodopa dose at MIBG evaluation, use of dopamine agonist at MIBG evaluation, severity and frequency of falls, and follow-up period after MIBG evaluation. Disease duration was defined as the period from the onset of motor symptoms to the time of MIBG evaluation. Severe falls were defined by the need for medical care after injuries. Injuries due to falls and the number of subjects with injury were summarized in the Supplementary Table 1. The present study protocol (number 3118) was approved by the local ethical committee at Tokushima University Hospital in March 2018.

### MIBG Imaging

MIBG imaging was performed 15 min (early) and 3–4 h (delayed) after intravenous injection of ^123^I-MIBG (111 mBq). The H/M ratio was calculated according to the standard protocol, as previously described (9).

### Statistical Analysis

All comparisons between the two groups were performed using Mann-Whitney *U*-tests and Fisher's exact probability tests for continuous and categorical variables, respectively. Jonckheere-Terpstra Test was performed to clarify the relationship between MIBG uptake and frequency of falls. For trend analysis, the subjects were divided into three groups according to MIBG uptake (group 1, delayed H/M ratio < 1.8; group 2, 1.8 ≤ delayed H/M ratio ≤ 2.7; group 3, 2.7 < delayed H/M ratio). Analyses were performed using SPSS statistics software (IBM; Armonk, NY). The predictive power of MIBG cardiac scintigraphy data for falls was evaluated using Kaplan-Meier analysis. Survival curves were compared using the log-rank test. This analysis was computed using R software (http://www.r-project.org/). *P* < 0.05 were considered statistically significant.

## Results

**Figure 1:**
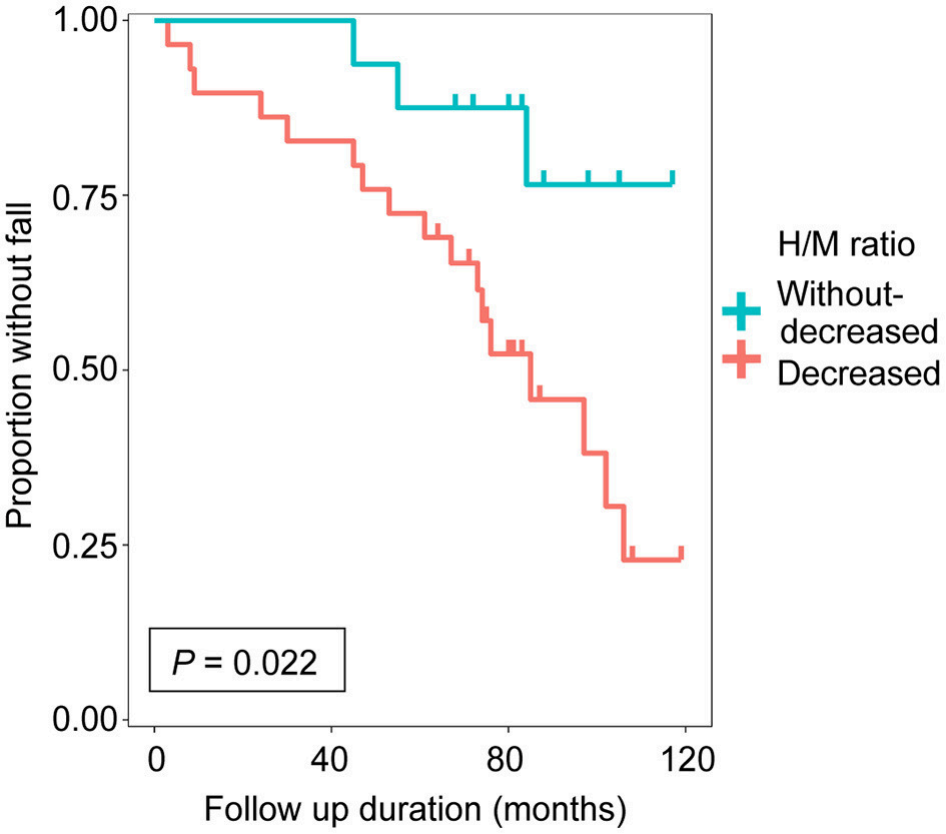
Cumulative fall-free survival based on delayed heart-to-mediastinum ratio (H/M ratio) was shown. The group with decreased uptake of ^123^I-meta-iodobenzylguanidine (MIBG) (*n* = 29, delayed H/M ratio < 1.8) and the group without decreased uptake of MIBG (*n* = 16, delayed H/M ratio ≥ 1.8).

We enrolled 45 patients with PD in this study based on the inclusion criteria. Each step of the recruitment process is presented in Supplementary Figure 1. The patient characteristics are summarized in Table 1. There were no significant differences in sex, age at onset, age at MIBG evaluation, Hoehn-Yahr stage, follow-up period, daily levodopa dose at MIBG evaluation, or use of dopamine agonist at MIBG evaluation between the groups. The group with decreased uptake of MIBG had a higher incidence of falls than the group without decreased uptake of MIBG (*P* = 0.022, log-rank test; Figure 1). Next, we investigated the effect of MIBG uptake on severity and frequency of falls. There was no significant difference in the incidence of severe falls between groups divided by MIBG uptake (*P* = 0.081, log-rank test; Supplementary Figure 2A). Frequency of falls was significantly associated with MIBG uptake in the delayed phase (*P* = 0.017, Jonckheere-Terpstra test; Supplementary Figure 2B).

**Table 1:** Characteristics of patients with PD in the study.

**Characteristics**	**Decreased uptake of MIBG (*n* = 29)**	**Without decreased** **uptake of MIBG (*n* = 16)**	***P***
Male (*n*)	17	9	0.562
Age at onset	62.7 (9.9)	58.8 (15.5)	0.307
Age at evaluation	67.7 (9.0)	61.3 (15.1)	0.132
Disease duration (*m*)	49.5 (52.6)	31.7 (32.7)	0.203
Hoehn-Yahr stage	1.95 (0.81)	1.77 (0.64)	0.357
Follow-up period (*m*)	89.6 (16.4)	87.8 (19.2)	0.669
Daily levodopa dose (mg)	141.4 (180.3)	82.3 (106.5)	0.188
Use of dopamine agonist (*n*)	13	5	0.286
Early H/M ratio	1.63 (0.25)	2.52 (0.47)	^*^ < 0.001
Delayed H/M ratio	1.35 (0.22)	2.60 (0.79)	^*^ < 0.001

*The results are shown as mean ± standard deviation*.

* *Indicates a significant difference between the group with decreased uptake of MIBG and that without decreased uptake of MIBG*.

*PD, Parkinson's disease; MIBG, ^123^I-meta-iodobenzylguanidine; n, number; m, months; H/M, heart-to-mediastinum*.

## Discussion

An MIBG-defined imaging phenotype was demonstrated to predict falls in patients with PD in the present study. This supports the potential utility of MIBG as an objective biomarker for prognosis of motor function.

The ideal biomarker is based on a risk factor that is a continuous variable and easy to measure across institutes. MIBG scintigraphy may be an easy and objective biomarker to predict falls, as uptake is standardized using a phantom in this technique. Another candidate biomarker is the β-amyloid 42 level in cerebrospinal fluid, which is associated with gait progression (10). A multifactorial model including MIBG uptake and β-amyloid 42 concentration could lead to development of a biomarker for prediction of falls.

Reduced MIBG uptake has been reported to be related to dementia and hallucination (6, 11). Motor dual-tasking deficits may predict falls (12), and cholinesterase inhibitors, which are used to treat cognitive impairment, have been reported to improve gait stability (2). Taken together, multiple lines of evidence suggest the presence of a significant association between cognitive function and falls. Considering that both regular falls and cognitive impairment may determine time to death (13), reduced MIBG uptake might predict an unfavorable prognosis in PD. Decreased cardiac uptake of MIBG reflects degeneration of postganglionic presynaptic nerve terminals in the adrenergic nervous system, where the presence of alpha-synuclein aggregates has been pathologically confirmed (14). It is reasonable that orthostatic hypotension is implicated in falls, and that falls are alleviated by droxidopa, which is a prodrug of norepinephrine (3). The relationship between MIBG scintigraphy and falls is understandable, as reduced uptake of MIBG reflects autonomic dysfunction, including orthostatic hypotension. Although MIBG uptake has been reported to be heterogeneous in PD (7), a significant reduction in MIBG uptake might indicate that alpha-synuclein aggregates are distributed widely in the whole body, including the brain. That said, in terms of pathology, decreased uptake of MIBG might be considered as a poor prognostic factor, which was further supported by clinical evidence that decreased uptake of MIBG was associated with cognitive impairment, hallucination, autonomic dysfunction and REM sleep behavior disorder (15). This study adds falls to the above-mentioned list.

The most important subtype of falls from the viewpoint of prognosis is thought to be recurrent/regular falls. Falls have been classified into two subtypes: falling forward and falling backward or sideways. The subtypes are thus based on the direction of the fall, which is reflective of the mechanism underlying each type of fall (16). However, it remains unknown whether there are differences in MIBG uptake and other factors between these subtypes.

The limitations of our study are moderate number of subjects and the use of univariate analysis due to lack of several key factors including cognitive assessment, autonomic function test, gait freezing, motor score, direction of the fall and the relation between falls and drugs, which may have biased the results. Future investigation will be carried out in a prospective cohort using multivariate analysis. This will allow us to adjust for confounders, such as age, sex, disease duration, disease severity, levodopa equivalent dose, mood, hallucination, dementia, and types of falls.

In summary, MIBG scintigraphy may predict motor prognosis as well as cognitive prognosis in patients with PD. Further prospective studies are needed to validate the present findings using multivariate analysis.

## Ethics Statement

This study was retrospective cohort study where the data were produced as a part of standard patient care. In accordance with the Ethics Committee of the Tokushima University Hospital, written informed consent was not required.

## Author Contributions

WS conceived the idea for this research. NM and WS designed the experiments. WS, SH, TF, YI, and RK recruited the patients. NM, WS, and SH analyzed the data. WS and NM wrote the first draft of the manuscript, with important contributions from YO, HO, YI, MH, and RK. All authors provided input for the final manuscript.

### Conflict of Interest Statement

The authors declare that the research was conducted in the absence of any commercial or financial relationships that could be construed as a potential conflict of interest.

## Supplementary Material

Supplementary Figure 1 A flow diagram for the present study was shown. The diagram illustrates the inclusion and exclusion criteria used. Data from forty-five subjects with Parkinson's disease were analyzed. PD, Parkinson's disease; MIBG, ^123^I-meta-iodobenzylguanidine.

Supplementary Figure 2 Cumulative severe fall-free survival based on delayed heart-to-mediastinum ratio (H/M ratio) was shown **(A)**. The group with decreased uptake of ^123^I-meta-iodobenzylguanidine (MIBG) (*n* = 29, delayed H/M ratio < 1.8) and the group without decreased uptake of MIBG (*n* = 16, delayed H/M ratio ≥ 1.8). The relationship between delayed H/M ratio and frequency of falls were shown **(B)**. The subjects were divided into three groups according to MIBG uptake (group 1, delayed H/M ratio < 1.8; group 2, 1.8 ≤ delayed H/M ratio ≤ 2.7; group 3, 2.7 < delayed H/M ratio).

Supplementary Table 1 Injuries due to falls and the number of subjects.
