# Supplementary figures and images for: Potential Utility of 123I-MIBG Scintigraphy as a Predictor of Falls in Parkinson's Disease
Source: Front Neurol. 2019 Apr 12;10:376. doi: 10.3389/fneur.2019.00376 (PMC6473994; doi:10.3389/fneur.2019.00376)

# Flow diagram for the present study

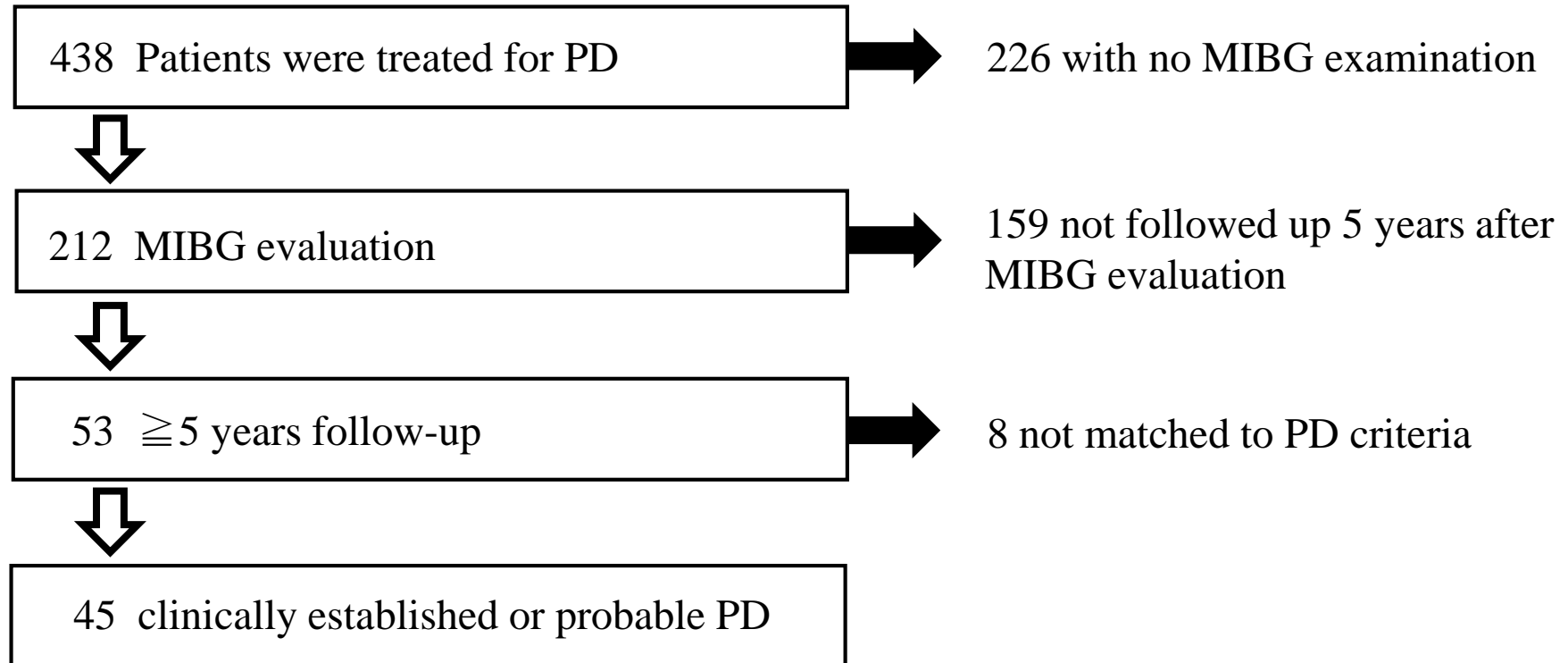

Supplement: Supplementary Figure 1 — A flow diagram for the present study was shown. The diagram illustrates the inclusion and exclusion criteria used. Data from forty-five subjects with Parkinson's disease were analyzed. PD, Parkinson's disease; MIBG, 123I-meta-iodobenzylguanidine. [file Image_1.pdf]

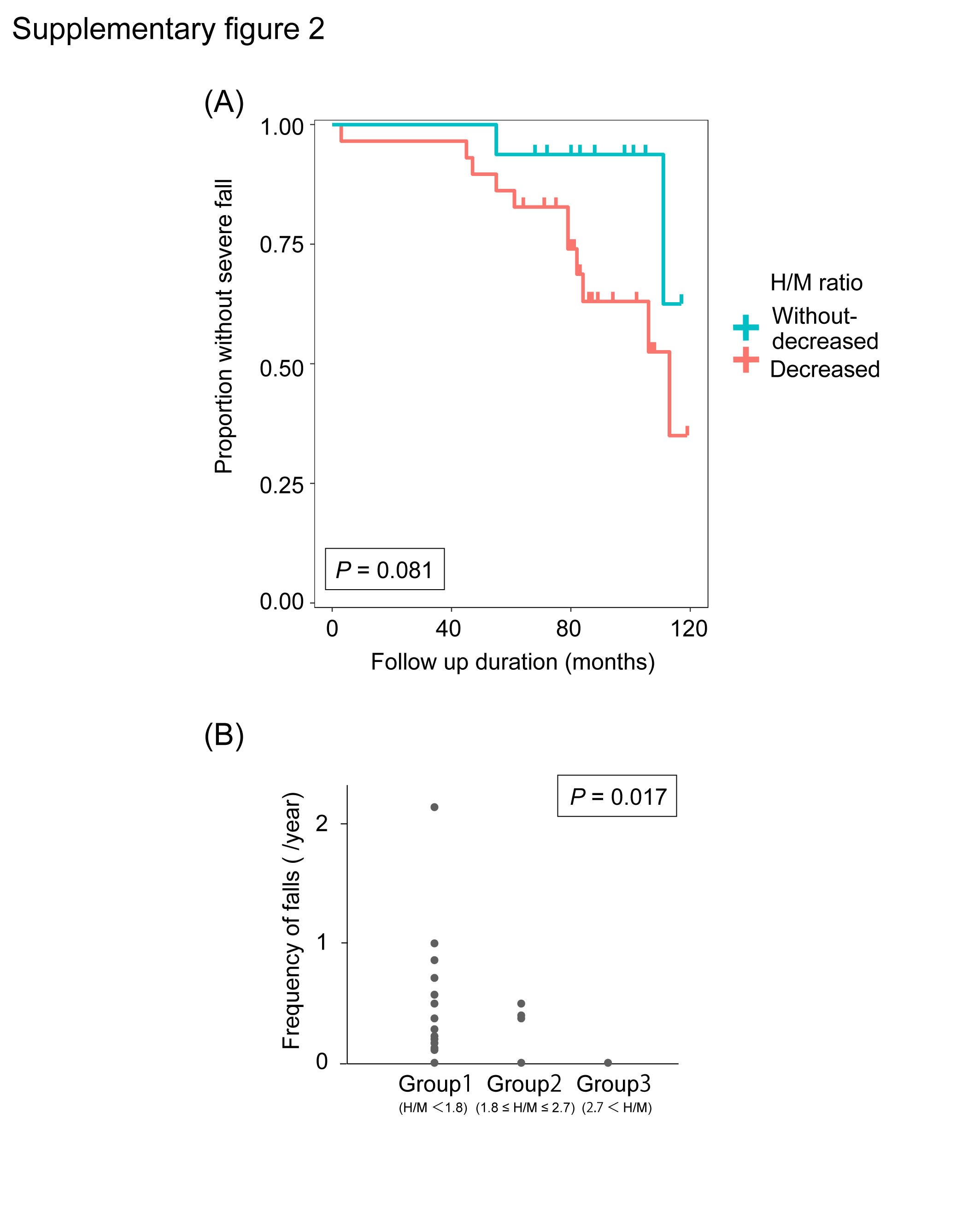

Supplement: Supplementary Figure 2 — Cumulative severe fall-free survival based on delayed heart-to-mediastinum ratio (H/M ratio) was shown (A). The group with decreased uptake of 123I-meta-iodobenzylguanidine (MIBG) (n = 29, delayed H/M ratio < 1.8) and the group without decreased uptake of MIBG (n = 16, delayed H/M ratio ≥ 1.8). The relationship between delayed H/M ratio and frequency of falls were shown (B). The subjects were divided into three groups according to MIBG uptake (group 1, delayed H/M ratio < 1.8; group 2, 1.8 ≤ delayed H/M ratio ≤ 2.7; group 3, 2.7 < delayed H/M ratio). [file Image_2.tif]
