# Supplementary material for: Potential Utility of 123I-MIBG Scintigraphy as a Predictor of Falls in Parkinson's Disease
Source: Front Neurol. 2019 Apr 12;10:376. doi: 10.3389/fneur.2019.00376 (PMC6473994; doi:10.3389/fneur.2019.00376)
Supplement: Supplementary Table 1 — Injuries due to falls and the number of subjects. [file Table_1.docx]

**Supplementary Table 1:**

Injuries due to falls and the number of subjects

| Injuries due to falls | The number of subjects |
| --- | --- |
| Bone fracture | 7 |
| Cut wound | 2 |
| Concussion | 1 |
| Dislocation of shoulder | 1 |
| Cervical disc herniation | 1 |
| Another | 1 |
